# Accelerated isotropic resolution 3D image-based navigators for coronary MR angiography

**DOI:** 10.1186/1532-429X-16-S1-P380

**Published:** 2014-01-16

**Authors:** Nii O Addy, Jieying Luo, R Reeve Ingle, Bob S Hu, Dwight G Nishimura

**Affiliations:** 1 Electrical Engineering, Stanford University, Stanford, California, USA; 2 Palo Alto Medical Foundation, Palo Alto, California, USA

## Background

Motion remains a primary challenge for MR coronary angiography. In our previous protocol, we performed retrospective 3D motion correction based on a set of orthogonal 2D image-based navigators (iNAV) [1]. Recent work examined the use of anisotropic-resolution 3D Cartesian iNAVs every heartbeat [2]. Capitalizing on the efficiency of non-Cartesian imaging and iterative reconstruction, we sought an improved 3D iNAVs acquisition with isotropic resolution, to facilitate whole-heart motion correction with translational or more advanced models. In this work, we propose a method providing 3D motion correction on a per-heartbeat basis using a variable-density 3D cones iNAV acquisition [3].

## Methods

Imaging was performed on a GE Signa 1.5 T Excite scanner with an 8-channel cardiac coil. Scans were acquired over a 28 × 28 × 14 cm3 FOV using an ATR-SSFP sequence and 3D cones trajectory [1]. The pulse sequence was modified by replacing the two 2D iNAVs with a single 3D cones acquisition collected after the last cardiac phase within the ATR-SSFP train. Due to the limited time window for the 3D iNAV, an undersampled, variable-density trajectory was used. At 4.38 mm isotropic resolution, a fully sampled 3D cones acquisition would require 290 readouts. However, using a variable-density design, decreasing the sampling density from 1.0 at the k-space origin to 0.26 at kmax, a 32-readout trajectory was achieved corresponding to an acceleration factor of 9 and acquisition time of 175 ms. 3D iNAVs were first reconstructed with gridding which served as a starting point for ESPIRiT [4]. ESPIRiT used a single set of coil sensitivities derived from a central calibration region from the fully sampled 3D cones imaging data. 3D motion information was extracted with the Insight Toolkit [5], using mutual information as the metric. Whole-heart images with 1.25 mm isotropic resolution were reconstructed with gridding. To test the feasibility of the 3D iNAVs, translational motion correction was applied using linear phase modulation [1].

## Results

**Figure 1:**
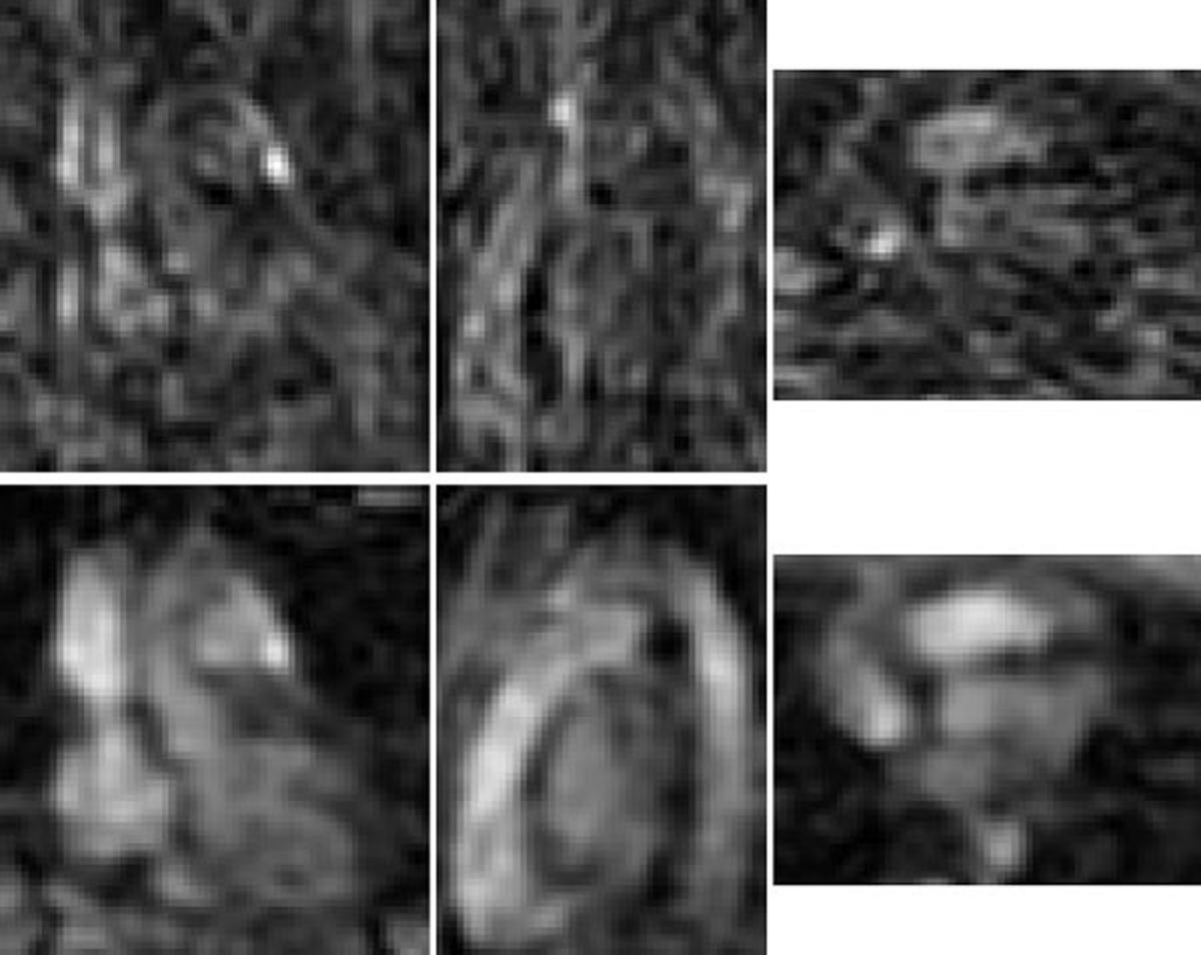
**A single heartbeat 3D iNAV reconstructed with gridding (top) and ESPIRiT (bottom) displayed in coronal (left), sagittal (middle), and axial (right) planes**.

**Figure 2:**
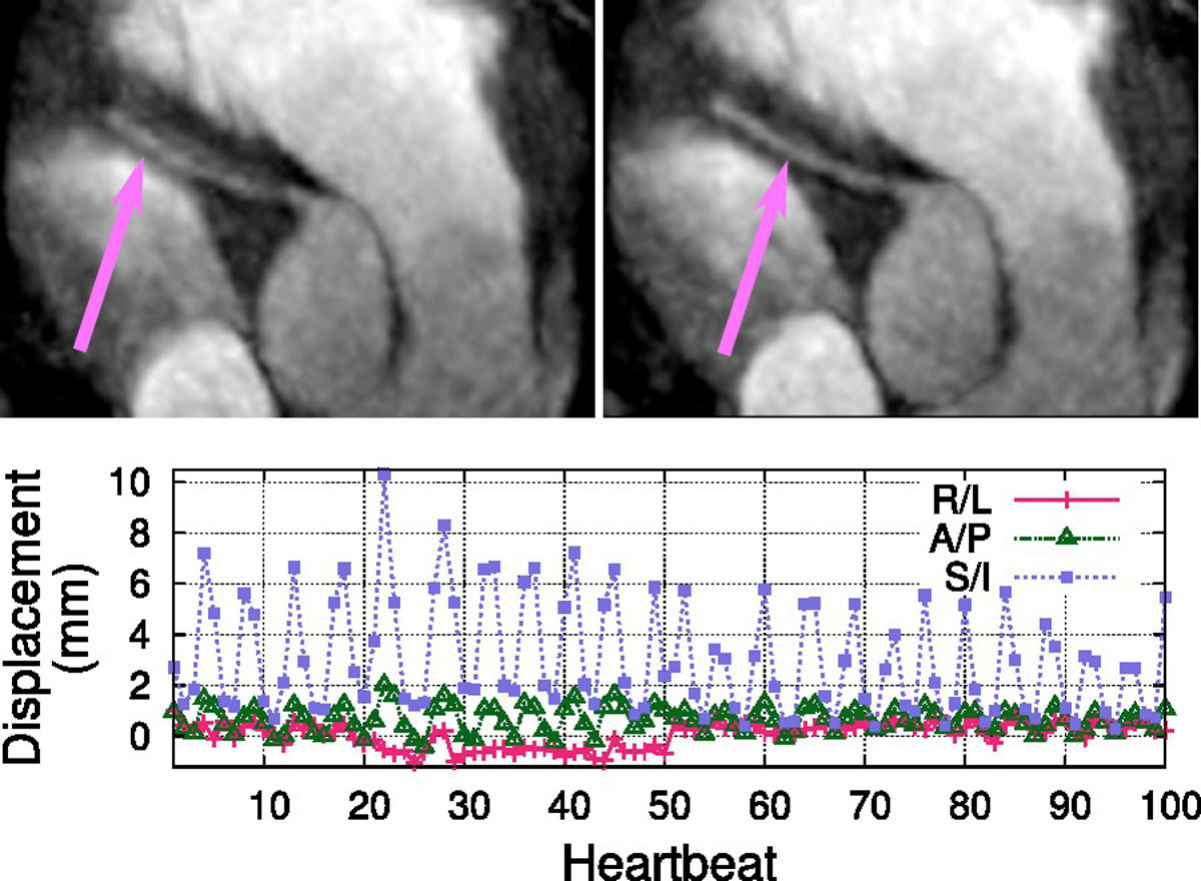
**Uncorrected (left) and corrected images (right) images of the right coronary artery**. Translation motion information from the first 100 heartbeats shown on bottom.

Figure 1 shows the initial 3D iNAV reconstruction with gridding and the corresponding reconstruction with ESPIRiT. ESPIRiT significantly reduced the aliasing artifacts, revealing contrast and features useful for motion estimation. Figure 2 shows motion estimates derived from the 3D iNAVs, in this case translational, and the resulting sharpening of the right coronary artery after motion correction.

## Conclusions

Acquiring 3D iNAVs every heartbeat, 3D motion of the heart was measured during a free-breathing coronary MRA acquisition to provide 100% respiratory efficiency and retrospective motion correction. Future work includes applying more advanced models based on the 3D iNAVs for improved correction.

## Funding

NIH T32 HL007846 NIH R01 HL039297 GE Healthcare.
